# Hypodermic Injection in Hernia

**Published:** 1877-05

**Authors:** 


Hypodermic Injections in Hernia.—Reporting upon three cases communicated to the *Societe de Chirurgie,* in which strangulated inguinal hernia was easily reduced after the hypodermic injection of morphia, M. Le Dentu observes that in these cases the injections certainly assisted their reduction, but it is doubtful how far they would have succeeded had the strangulation been more decided and of longer duration. If the surgeon is called to the case immediately, the injection may be of use by dissipating the pain and spasm; but if some hours have elapsed, it will be always of less value than chloroform, which enables us to at once recognize whether the hernia is reducible or the operation necessary.—*Med. Times and Gaz.*, April 8, 1877, from *Gaz. des Hop.*, March 31.

